# Fatty Acids of CLA-Enriched Egg Yolks Can Induce Transcriptional Activation of Peroxisome Proliferator-Activated Receptors in MCF-7 Breast Cancer Cells

**DOI:** 10.1155/2017/2865283

**Published:** 2017-03-26

**Authors:** Aneta A. Koronowicz, Paula Banks, Adam Master, Dominik Domagała, Ewelina Piasna-Słupecka, Mariola Drozdowska, Elżbieta Sikora, Piotr Laidler

**Affiliations:** ^1^Department of Human Nutrition, Faculty of Food Technology, University of Agriculture in Krakow, Balicka 122, 30-149 Krakow, Poland; ^2^Department of Biochemistry and Molecular Biology, Medical Centre for Postgraduate Education, Marymoncka 99, 01-813 Warsaw, Poland; ^3^Department of Medical Biochemistry, Jagiellonian University Medical College, Kopernika 7, 31-034 Krakow, Poland

## Abstract

In our previous study, we showed that fatty acids from CLA-enriched egg yolks (EFA-CLA) reduced the proliferation of breast cancer cells; however, the molecular mechanisms of their action remain unknown. In the current study, we used MCF-7 breast cancer cell line to determine the effect of EFA-CLA, as potential ligands for peroxisome proliferator-activated receptors (PPARs), on identified in silico PPAR-responsive genes:* BCAR3*,* TCF20*,* WT1*,* ZNF621*, and* THRB* (transcript TR*β*2). Our results showed that EFA-CLA act as PPAR ligands with agonistic activity for all PPAR isoforms, with the highest specificity towards PPAR*γ*. In conclusion, we propose that EFA-CLA-mediated regulation of PPAR-responsive genes is most likely facilitated by* cis9,trans11CLA* isomer incorporated in egg yolk. Notably, EFA-CLA activated PPAR more efficiently than nonenriched FA as well as synthetic CLA isomers. We also propose that this regulation, at least in part, can be responsible for the observed reduction in the proliferation of MCF-7 cells treated with EFA-CLA.

## 1. Introduction

Peroxisome proliferator-activated receptors (PPARs) are ligand-activated transcription factors. Various fatty acids and their metabolic derivatives act as natural ligands for PPARs [1]. Some, including linoleic, linolenic, and arachidonic acid, were found to activate PPARs even at micromolar, physiologically relevant concentrations [2]. Hydroxyoctadecadienoic acids (HODEs), products of linoleic acid oxidation as well as arachidonic acid metabolite 15d-PGJ2 (15-deoxyprostaglandin J2), were also associated with PPAR activation [3, 4].

It has been suggested that ligand-dependent activation of PPARs results in the inhibition of proliferation in some model cancer cell lines [5–7]. In particular, PPAR*γ* isoform was shown to reduce cancer cell proliferation as well as regulate cell differentiation, activate apoptosis, and inhibit angiogenesis [8–10]. Specifically, the administration of specific PPAR*γ* agonist resulted in cells arrest in G1 phase and inhibited proliferation [5, 11]. However, available literature presents also contradicting results. In some studies, PPAR*γ* specific antagonist, T0070907, significantly reduced proliferation and migration of breast cancer cells [12, 13].

Conjugated linoleic acid (CLA) term includes several isomers of linoleic acid, with two main isomers:* cis9,trans11* (80–90% of total CLA) and* trans10,cis12*. Available literature shows that CLA acts as a potent PPARs ligand and is involved in modulating lipid metabolism through PPAR-mediated pathways [14]. However, data showed isomer-specific activity of CLA; specifically,* cis9,trans11* was characterized as PPAR agonist [15, 16] while* trans10,cis12* was shown to inhibit the activity of synthetic PPAR agonists [15]. In addition, studies showed potential antitumor properties of* cis9,trans11* [17–20] while the opposite effect was observed for* trans10,cis12* isomer [18].

PPARs act as transcription factors and regulate the expression of dependent genes by binding to their PPREs. A significant number of genes regulated by PPARs have been described; however, the list is not exhaustive and is constantly being updated as new results are being published from both experimental data and bioinformatics analyses of promoter regions and PPRE consensus sequences. In the current study, we applied those tools to identify in silico PPRE selected genes involved in cell cycle progression and proliferation. Next, we analyzed the effect of synthetic* cis9,trans11CLA* and* trans10,cis12CLA* isomers as well as a mixture of fatty acids extracted from CLA-enriched and nonenriched egg yolk on the expression of those genes. To the best of our knowledge, our study is the first to address the effect of CLA incorporated in fatty acids profile of the egg yolk; we expect that activity of CLA in such a “bioorganic” form may deviate from that of a synthetic form. The presence of other fatty acids in an egg yolk, which themselves can act as potential ligands for PPARs, may modulate the action of CLA; therefore, our data may be particularly important for the evaluation of CLA-enriched food products.

## 2. Materials and Methods

### 2.1. Production of CLA-Enriched Egg Yolks

Production of CLA-enriched egg yolks was performed in the National Research Institute of Animal Production in Krakow (Poland), as per the recommendations of the Local Animal Ethics Committee (approval number: 851/2011) as described previously [21]. Eggs were collected and stored at 4°C, and yolks were separated from albumen, homogenized, and frozen at −20°C. Samples were then lyophilized and again stored at −20°C until further analyses.

### 2.2. Extraction and Analysis of Fatty Acids Composition

Lipids from control and CLA-enriched yolks were extracted by using modified Folch method [22] as described previously [23]. 10 mg of each lipid extract was subjected to saponification with 0.5 M KOH/methanol followed by methylation with 14% (v/v) BF3/methanol and extraction with hexane. Fatty acid methyl esters (FAME) were analyzed by GC/MS as described previously [23].

### 2.3. CLA Isomers and Agonists/Antagonists of PPAR

*cis9,trans11CLA* and* trans10,cis12CLA* isomers (Nu-Chek Prep, USA) were dissolved in ethanol and stored under nitrogen in −20°C and were introduced to cell cultures at final concentrations corresponding to their concentration in CLA-enriched egg yolk:* cis9,trans11* at 30 *μ*M and* trans10,cis12* at 12 *μ*M.

The synthetic agonists and antagonists for PPAR*α* (WY14643 and GW-6471), PPAR*δ* (GW-0742 and GSK0660), and PPAR*γ* (pioglitazone (PIO), troglitazone, and T0070907) were prepared as per appropriate protocols of the manufacturer. Respective concentrations were selected based on their EC/IC50 characteristics and confirmed for MCF-7 cell line using Cytotoxicity LDH Test (Roche, Poland).

### 2.4. Cell Cultures

The human breast adenocarcinoma cell line MCF-7 (ATCC® HTB­22TM) was purchased from the American Type Culture Collections. Cells were cultured in appropriate medium (Sigma-Aldrich, MO, USA) as per the ATCC protocol with the addition of 10% FBS (Sigma-Aldrich, MO, USA).

Cell viability was determined by Crystal Violet Assay (Sigma-Aldrich, MO, USA).

### 2.5. Fatty Acid Treatment

The experimental medium contained MEM supplemented with 10% FBS and appropriate treatment: (a) fatty acids extract at 0.5 mg/mL from CLA-enriched egg yolks (EFA-CLA), (b) fatty acids extract at 0.5 mg/mL from nonenriched egg yolks (EFA), (c)* cis9,trans11* synthetic isomer (final concentration at 35 *μ*M), (d)* trans10,cis12* synthetic isomer (final concentration at 13 *μ*M), (e) untreated cell control (empty control, EC), and (f) negative control (NC; ethanol at final concentration 0.1%). Synthetic PPARs agonists and antagonist were used as positive controls for PPAR*α* (10 *μ*M WY14643 and 10 *μ*M GW-6471), PPAR*δ* (2 *μ*M GW-0742 and 1 *μ*M GSK0660), and PPAR*γ* (40 *μ*M PIO, 10 *μ*M troglitazone, and 10 *μ*M T0070907). Each treatment included 3 biological and 3 technical replicates.

### 2.6. Plasmids

PPAR expression vectors were prepared using Gateway® Cloning System (Thermo Fisher, USA). Briefly, PPARA (CR456547_1), PPARD (NM_006238.4), and PPARG (NM_015869.4) ORF sequences were synthesized, optimized for the expression in human cells, and cloned into the pDONR221 Entry Vectors (GeneArt, Thermo Fisher, USA). Subsequently, the ORF inserts were transferred into pcDNA6.2/N-EmGFP-DEST Destination Vectors (Thermo Fisher, USA) under the CMV promoter control via Clonase II Recombination Reaction.

### 2.7. Cell Transfection with PPAR Encoding Plasmids

Cell lines with PPARA, PPARD, and PPARG overexpression were obtained via transient transfections with pcDNA6.2/N-EmGFP-DEST vectors containing respective human PPAR ORF. MCF-7 cells were seeded on 12-well plates, at 1 × 10^5^ cells per well. 24 h after seeding, cells were transiently transfected with 1.5 *μ*g of PPAR encoding plasmids using Lipofectamine (Thermo Fisher Scientific, MA, USA) in OPTI-MEM medium (Thermo Fisher Scientific, MA, USA). 24 h after transfection, the growth medium was replaced with selective MEM medium with 10% FBS and 5.0 *μ*g/mL blasticidin (BioShop, Canada). Transfected cells were cultured until confluency.

Real-time PCR and western blot method were performed to confirm the presence of PPAR plasmids after transfection (Figure S1 and Table S2, Supplementary Material available online at https://doi.org/10.1155/2017/2865283).

### 2.8. Transfection with PPRE Plasmid

Cell lines overexpressing, respectively, PPARA, PPARD, and PPARG were seeded on the 12-well plates, at 1 × 10^5^ cells per well. After 24 hours, cells were transfected with 0.7 *μ*g X3 PPRE-TK-luc plasmid (Cat. # 1015, Addgene, USA) and 0.7 *μ*g pRL control (Cat. # E2261, Promega, WI, USA) using Lipofectamine (Thermo Fisher Scientific, MA, USA) in OPTI-MEM medium (Thermo Fisher Scientific, MA, USA).

### 2.9. Dual-Luciferase Assay

24 hours after transfection with PPRE plasmid, the medium was again replaced with MEM medium containing 10% FBS and appropriate experimental treatment as described above. 24 hours after treatment, cells were harvested for isolation of protein luciferase.

The luciferase protein (*Photinus pyralis *and* Renilla reniformis*) detection was performed using Dual-Luciferase® Reporter Assay System (Promega, WI, USA) in GloMax® 20/20 Single Tube Luminometer (Promega, WI, USA), according to the manufacturer's instructions.

### 2.10. In Silico Selection and Experimental Confirmation of PPAR-Dependent Genes (PPAR-Responsive mRNAs)

PPAR-responsive genes were selected in silico by searching for peroxisome proliferator hormone response elements (PPREs, AGGTCANAGGTCA) within promoters and/or 5′-*cis*-regulatory regions of the promoters of genes involved in cell cycle progression and proliferation. This search was performed with NCBI Gene and Blast tools.

Experimentally, 24 hours after transfection with respective PPAR plasmids, the medium was replaced with MEM medium containing 10% FBS and appropriate experimental treatment as described above. 48 hours after treatment, cells were harvested for mRNA isolation and RT-qPCR.

### 2.11. RNA Isolation, cDNA Synthesis, and RT-qPCR Analysis

Total RNA was isolated from the cells using RNA isolation kit for cell cultures (A&A Biotechnology, Poland). Reverse transcription was performed on 1 *μ*g of total RNA using Maxima First-Strand cDNA Synthesis kit for RT-qPCR (Thermo Scientific, MA, USA). Quantitative verification of genes was performed using CFX96 Touch™ Real-Time PCR Detection System instrument (Bio-Rad, CA, USA) and SYBR Green Precision Melt Supermix kit (Bio-Rad, CA, USA). Conditions of individual PCR reactions were optimized for given pair of oligonucleotide primers (Table S1, Supplementary Material). Basic conditions were as follows: 95°C for 10 min, 45 PCR cycles at 95°C, 15 s; 59°C, 15 s; 72°C, 15 s, followed by melting curve analysis (65–97°C with 0.11°C ramp rate and 5 acquisitions per 1°C). Results were normalized using at least two reference genes (*GAPDH*,* HPRT1*,* ACTB*, or* HSP90AB1*) and were calculated using the 2^−ΔΔC^T method [24].

### 2.12. Protein Isolation and Western Blot Analysis

Cell lysis was carried out using Cell Lysis Buffer (Cell Signaling Technology, MA, USA) as per the manufacturer's protocol. Total protein quantification was performed using Pierce BCA™ Protein Assay Kit (Thermo Fisher Scientific, MA, USA).

Each western blot followed a similar procedure. Protein extract was separated on a polyacrylamide gel and transferred to a nitrocellulose filter (Bio-Rad, CA, USA) by wet electroblotting. Subsequently, the immobilized proteins were incubated with appropriate primary antibody, specific for PPAR*α* (SAB2101852), PPAR*γ* (SAB2101853), and PPAR*δ* (AV32880) as well as for selected in silico WT1 (SAB2102716), THRB (AV36994), and TCF20 (SAB2106444) from Sigma-Aldrich, MO, USA, or *β*-actin (#8457) or *β*-tubulin (#2128) from Cell Signaling Technology, MA, USA. Finally, appropriate secondary antibody conjugated with horseradish peroxidase (#7074, Cell Signaling Technology, MA, USA) was applied. Detection was executed by chemiluminescence, using Clarity™ Western ECL Substrate (Bio-Rad, CA, USA). To remove the antibodies from the membrane, we used western blot stripping buffer (Thermo Scientific, MA, USA).

### 2.13. Statistical Analysis

All experiments were performed at least three independent times and measured in triplicate. Shapiro-Wilk's test was applied to assess normality of distribution. An independent samples *t*-test was applied to compare unpaired means between two groups. *p* < 0.05 was considered statistically significant. All analyses were performed using Statistica ver.12 (StatSoft, Tulsa, OK, USA).

## 3. Results

### 3.1. Cell Viability

**Figure 1:**
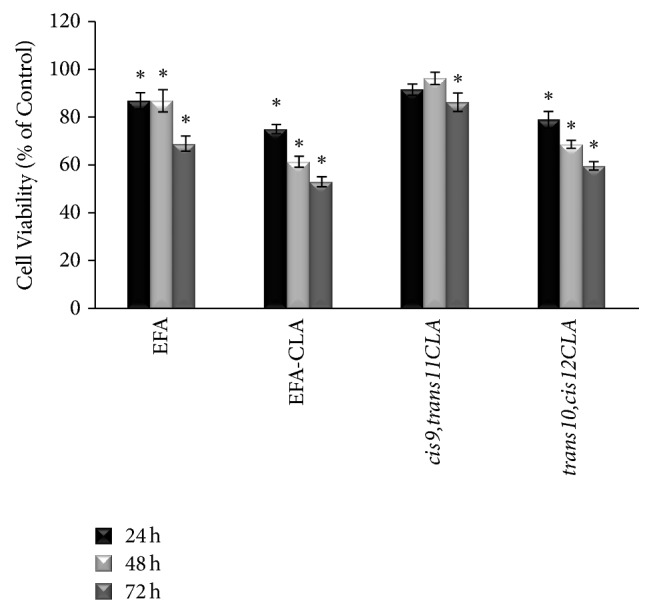
Effect of fatty acids on MCF-7 cells viability. Values are expressed as means ± SD for *N* ≥ 9, standardized to control (NC) as 100%. Statistical significance was based on *t*-test; ^*∗*^*p* < 0.05 versus control.

Treatment with both extracts, EFA and EFA-CLA, decreased viability of MCF-7 breast cancer cell line compared to the control; however, the effect of EFA-CLA was more evident compared to EFA. 72 h after treatment, cell viability in EFA-CLA-treated group decreased by 50% while for EFA the decrease in viability reached 32% (Figure 1). Treatment with synthetic* trans10,cis12CLA* reduced cell viability in a linear manner with incubation time, reaching 43% at 72 h. The reductive effect of* cis9,trans11CLA* isomer was less evident and statistically significant only after 72 h (overall reduction in viability by 15%).

### 3.2. Effects of EFA-CLA on Transcriptional Activity of PPARs

**Figure 2:**
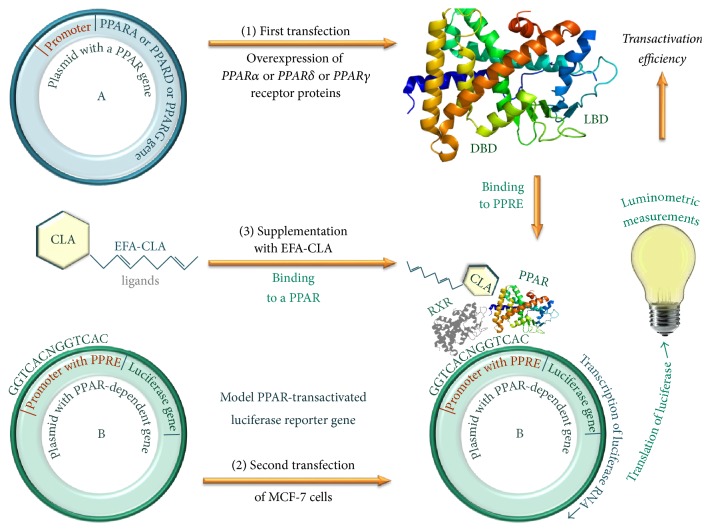
Experimental mechanism for studying the activity of EFA-CLA as a ligand for PPAR. DBD: DNA-binding domain specific for PPRE sequence in promoter regions of genes regulated by PPAR; LBD: ligand-binding domain (e.g., EFA-CLA).

**Figure 3:**
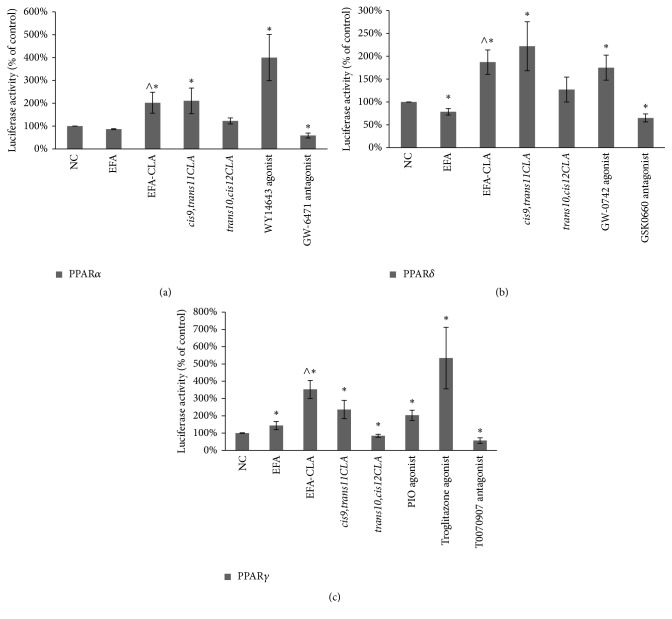
Effect of EFA-CLA on the activity of (a) PPAR*α*, (b) PPAR*δ*, and (c) PPAR*γ* based on measured luciferase activity in dual-luciferase assay.* Values are expressed as means ± SEM for N* ≥ 12*, standardized to control (NC) as 100%. Statistical significance was based on t-test*; ^*∗*^*p* < 0.05 versus* NC or *^∧^*p* < 0.05 versus* EFA*.

To analyze the activity and specificity of various CLAs as potential PPAR ligands, we applied the PPAR-dependent luciferase expression model (Figure 2). We used specific agonists and antagonists for each isoform of PPARs as positive controls. Our results confirmed the expected effects of selected agonists and antagonists (Figures 3(a)–3(c)). The effect of experimental FA extracts varied. Compared to the negative control, EFA-CLA significantly increased the activity of PPAR*α* (202% of NC; *p* < 0.05; Figure 3(a)), PPAR*δ* (187.10% of NC; *p* < 0.01; Figure 3(b)), and PPAR*γ* (353% of NC; *p* < 0.001; Figure 3(c)). Compared to EFA extract, EFA-CLA also showed statistically significant activation of all PPAR isoforms (Figures 3(a)–3(c)). Synthetic* cis9,trans11* isomer also activated significantly all PPARs, PPAR*α* (211% of NC; *p* < 0.05; Figure 3(a)), PPAR*δ* (221.88% of NC; *p* < 0.01; Figure 3(b)), and PPAR*γ* (237% of NC; *p* < 0.01; Figure 3(c)).* trans10,cis12CLA* isomer had little or no effect on the activation of PPAR*α* and PPAR*δ* (Figures 3(a) and 3(b)); however, it reduced the activity of PPAR*γ* (85% of NC; *p* < 0.05; Figure 3(c)).

### 3.3. Selective Effect of FA on Transcriptional Activity of PPARs

**Figure 4:**
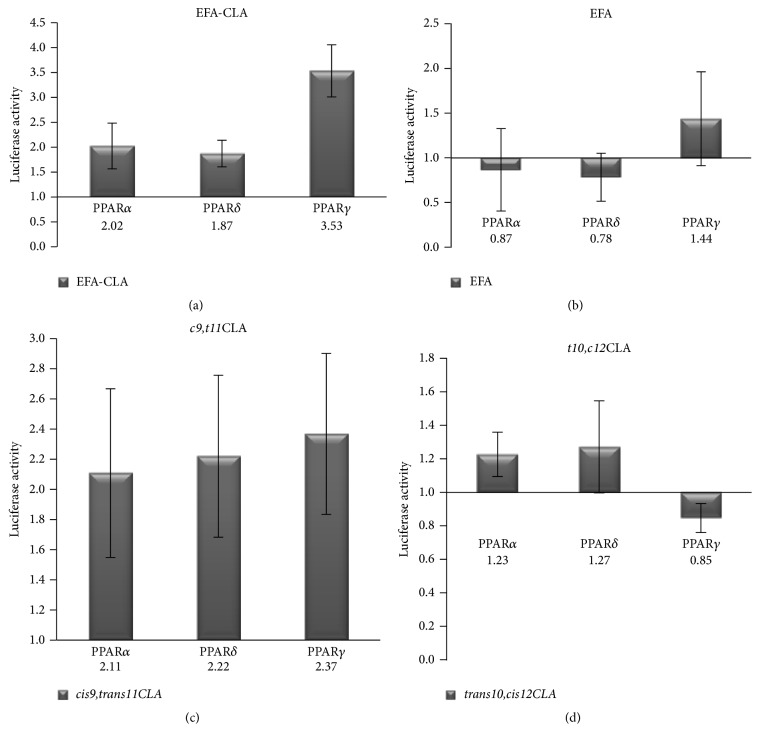
Selective effect of FA on PPARs expressed as fold difference versus control (100%), based on data from Figure 3. Values are expressed as means ± SEM for the *N* ≥ 12.

The selective effects of the studied FA as potential PPAR ligands are shown in Figures 4(a)–4(d). EFA-CLA was determined to be the most specific for PPAR*γ* (3.5-fold increase in activity, *p* < 0.001; Figure 4(a)). EFA extract acted as an antagonist towards both PPAR*α* and PPAR*δ*, while it exhibited only negligible agonist activity on PPAR*δ* (1.44-fold increase in activity, *p* > 0.05, Figure 4(b)).* cis9,trans11* isomer showed agonist properties towards all PPAR isoforms, with the strongest effect on PPAR*γ* (2.37-fold increase in activity, *p* < 0.005; Figure 4(c)).* trans10,cis12* isomer showed no significant effect on transactivation of both PPAR*α* and PPAR*δ* (*p* > 0.05, Figure 4(d)), while it showed an antagonist activity towards PPAR*γ* (*p* < 0.01, Figure 4(d)).

### 3.4. Prediction of Potential PPRE-Dependent Genes In Silico

**Figure 5:**
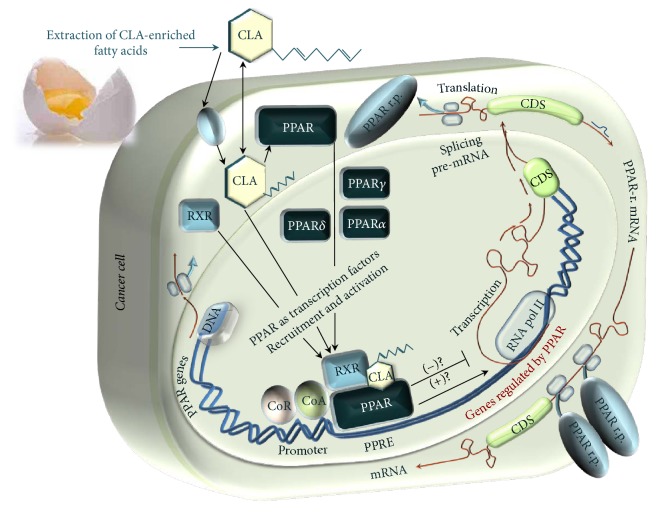
Molecular aspects of CLA-induced accumulation of PPAR-responsive transcripts. PPAR-r. mRNA: PPAR-regulated mRNAs; PPAR-r.p.: PPAR-regulated proteins; PPRE: peroxisome proliferator hormone response element (AGGTCANAGGTCA); RXR: retinoid X receptor; ORF: open reading frame (coding sequence).

The prediction of potential PPRE-responsive genes was performed in silico. NCBI database was searched for the presence of specific PPRE (peroxisome proliferator response element) consensus sequences (AGGTCAAAGGTCA, AGGTCAGAGGTCA, AGGTCACAGGTCA, or AGGTCATAGGTCA) in the 5′ region of genes linked to oncogenesis and cell cycle (Figure 5). Seven genes were identified:* BCAR3*,* LZTS*,* SLC5A1*,* TCF20*,* WT1*,* ZNF621*, and* THRB* (transcript TR*β*2), potentially regulated by PPARs (Table 1).* THRB* gene was identified by the presence of the PPRE consensus sequence in a region of the alternative promoter for TR*β*2 isoform (intron between the 4th and 5th exon). Among identified potential PPRE-dependent genes, few were selected for further experimental analyses, including* TCF20*,* WT1 ZNF621*, and* THRB*.

### 3.5. Effects of EFA-CLA on the Expression of PPAR-Regulated Genes

Expression of selected PPAR-responsive genes (containing PPRE) has been tested in response to various experimental fatty acids as potential ligands for PPARA, PPARD, or PPARG. Our results showed both agonist and antagonist effects of studied experimental FA.

EFA-CLA added to the PPAR*γ*-overexpressing cells elevated the expression of* TCF-20* over 3.2-fold and* ZNF621* over 3.1-fold, while decreasing the expression of* WT1* gene 1.2-fold. However, the latest may be explained, at least in part, from the fact that* WT1* gene is cotranscribed with interfering long, noncoding antisense RNA (WT1-AS) from the same bidirectional promoter. For cells overexpressing PPAR*δ*, EFA-CLA treatment resulted in the elevated expression of* TCF-20 *over 3-fold, while for the PPAR*α*-overexpressing cells* ZNF621* gene was upregulated 1.8-fold.

The strongest enhancement of* TCF-20 *expression (over 13-fold) was observed in PPAR*γ*- and PPAR*δ*-overexpressing cells after treatment with* trans10,cis12CLA*. Interestingly, the expression of* THRB *(TR*β*2 variant) gene was also strongly increased by the treatment with* trans10,cis12CLA* over 18.15-, 17.2-, and 7.9-fold in PPAR*δ*-, PPAR*γ*-, and PPAR*α*-overexpressing cells, respectively, but not observed for EFA-CLA-treated cells. Those results show that the presence of other fatty acids in EFA-CLA mixture contributes to the overall effect of FA treatment.

It is clear that the expression of the selected genes* (TCF-20*,* WT1*,* ZNF621*, and* THRB)*, which were identified for the first time in this work as putative PPAR-responsive genes, was altered in the presence of the used agents (Table 2) and that among them* TCF-20* was affected the most by EFA-CLA.

## 4. Discussion

Chicken egg enriched with conjugated linoleic acid (CLA) via feed modification meets the criteria of the functional food product. Based on Roberfroid's [25] classification, CLA-enriched egg can be considered as a conventional food product that is intended to be consumed as a part of a normal diet but is modified to contain biologically active substances, that is, CLA isomers. It has been shown to have a beneficial effect on physiological functions of the human body, in a way that goes beyond its nutritional value, specifically by lowering the risk of developing atherosclerosis [26]. Our previous studies showed additional beneficial properties of CLA-enriched eggs in reducing proliferation of breast cancer and melanoma cells [23, 27]. The current manuscript supports those findings as our new results showed that fatty acids extract from CLA-enriched egg yolks (EFA-CLA) reduced the viability of MCF-7 breast cancer cell line (Figure 1). However, the molecular mechanism is not fully understood. Comparison of the effect on cancer cell proliferation between extracts from CLA-enriched and nonenriched egg yolks could lead to the conclusion that it is simply the result of the presence of CLA isomers incorporated in the egg yolk lipids. Available literature would support such a hypothesis as numerous studies showed an inhibitory effect, especially for* cis9,trans11CLA* isomer, on tumor cells [28–32]. Indeed, our analysis of FA profile of CLA-enriched egg yolk showed that* cis9,trans11CLA *was incorporated more efficiently (3 : 1 ratio) than* trans10,cis12* isomer [21] and therefore could predominate in EFA-CLA. Interestingly, comparison of the effect of synthetic CLA isomers with CLA-EFA from egg yolk showed the advantage of the latter in reducing cancer cell viability (Figure 1). The analysis of fatty acids profiles between enriched and nonenriched egg yolks revealed not only CLA incorporation but also unexpected, significant change in SFA/MUFA ratio, specifically an increase in total SFA concentration at the expense of MUFA. Thus, a question arises of whether it is an individual or combined effect of CLA and modified SFA/MUFA ratio in enriched egg yolks on MCF-7 cell line [23]. We observed that results of CLA-EFA are most likely achieved by the effect of both: incorporated CLA isomers and other fatty acids in eggs modified organically through hens' diet [23]; however, this issue requires further research.

It has been shown that PPAR agonists have different properties for individual PPAR isoforms, with different absorption and distinctive gene expression profiles. To our knowledge, this is the first study focused on the effect of FA from CLA-enriched egg yolks on transcriptional activation of PPARs (PPAR*α*, PPAR*γ*, and PPAR*δ*). All experiments included as controls synthetic CLA isomers as well as standard agonists and antagonists of different PPARs. Our results showed that EFA-CLA extract exhibits the properties of agonists for all PPAR isoforms (Figures 3(a)–3(c)); however, those properties seem to be most selective towards PPAR*γ* (Figure 4). Interestingly, PPAR*γ* has been associated with the greatest impact on cancer cell proliferation, survival, and differentiation, and its ligands are associated with anticancer properties [33, 34]. In addition, as observed for EFA-CLA, transactivation of PPAR receptors is more effective compared to fatty acids extracted from a nonenriched egg yolk (EFA) (Figures 3(a)–3(c)). Since* cis9,trans11CLA* isomer showed PPAR agonist activity (Figures 3(a)–3(c)) and since this isomer was 3-fold more efficiently incorporated into egg yolks than* trans10,cis12CLA *[23], it could be hypothesized that* cis9,trans11CLA* plays a significant role in EFA-CLA-mediated activation of PPARs.

The effect of synthetic CLA isomers provided us with important information about their specificity. While* cis9,trans11* isomer acted as a PPAR agonist (Figures 3(a)–3(c)), the antagonist effect was observed for* trans10,cis12* isomer, specifically on PPAR*γ* (Figure 3(c)). Available literature is consistent with our results.* cis9,trans11* isomer has been reported to inhibit cell growth [15, 16] showing antitumor properties [17–20]. It has been found as well that the presence of* trans10,cis12* isomer may abrogate the antiproliferative activity of* cis9,trans11* [18] and even inhibit the activity of synthetic PPAR agonists [15]. Thus, it is even more interesting that our results showed more efficient reduction in cancer cells proliferation for EFA-CLA treatment than using a pure synthetic* cis9,trans11CLA* isomer that may suggest other factors including modified SFA/MUFA ratio in enriched egg yolks [23], supporting antiproliferative action of* cis9,trans11CLA* isomer.

PPARs act as transcription factors and regulate the expression of dependent genes by binding to their PPREs. Available literature gives a number of genes regulated by PPARs; the ligand-dependent transcription factors [35] and the expression of those genes can be both inhibited or activated depending on the ligand, suggesting selectivity [36]. CLA isomers have been found to act as PPAR ligands and shown to be involved in the inhibition of transcription of genes including* TNF* [37],* NFKB1* [38], and* NR1I3* [39] as well as transactivation:* TGFB1* [40],* BRCA1* [41],* PTEN* [42],* p21/WAF1/CDKN1A* [43],* CEBPA* [44],* ABCB4* [45], and* AOX *[46]. Although a significant number of genes regulated by PPARs have been described, the list is not exhaustive and is constantly updated as new results are being published from both experimental data and bioinformatics analyses of promoter regions containing PPRE consensus sequences (AGGTCANAGGTCA) (Figure 5).

In the current study, we applied bioinformatic tools to find genes with PPRE and analyze the effect of CLA on the expression of these genes. To our knowledge, we proposed several new genes that could be potentially PPAR-regulated:* BCAR3*,* LZTS*,* SLC5A*,* TCF20*,* WT1*,* ZNF621*, and* THRB* (transcript TR*β*2) (Table 2). Since preliminary data showed that some of them were strongly regulated by PPARs, we studied the expression of* TCF20*,* WT1*,* THRB (TRβ2)*, and* ZNF621 *genes in the context of various PPAR ligands, including EFA-CLA.

First one* TCF20* can act as a phosphoserine-specific repressor of estrogen receptors (ER) in estrogen-dependent tumors [47]. MCF-7 human breast carcinoma cell line is estrogen receptor (ER) positive; thus, the expression of* TCF20* should inhibit ER and consequently impair the viability of the tumor cells. Our results confirm these assumptions, showing elevated* TCF20* mRNA level in cells treated with EFA-CLA. This effect was much stronger than for EFA (Table 2). Interestingly, the most pronounced effect was found for* trans10,cis12CLA* isomer (Table 2), which may explain its advantages over the* cis9,trans11CLA* in reducing the viability of MCF-7 (positively correlates with its effect on the reduction in cell viability) (Figure 1). In contrast to Pariza et al. [18], this result also suggests that* trans10,cis12CLA* isomer could support antiproliferative action of* cis9,trans11CLA *in EFA-CLA via transcription-enhancing effects on* TCF20*.

Available literature addresses the relationship between receptors encoded by PPAR and* THRB* genes [48–50].* THRB* encodes three isoforms of human thyroid hormone receptor: TR*β*1 and tissue-specific TR*β*2 and TR*β*4, which are thought to be engaged in cell cycle control and metabolism [51]. Recently,* THRB* has been studied as a tumor suppressor [52]. Although TR*β*1 isoform has been found to play a role in the competitive inhibition of the PPAR transactivation [53], there is limited information on the relationships between TR*β*2 and PPAR receptors. TR*β* and PPAR receptors are linked by the same obligatory coreceptor, retinoid X receptor (RXR), that binds to their heterodimeric partners before binding to DNA. Although RXR plays a central role in regulating the activity of a number of nuclear hormone receptors including TR*β* and PPARs by acting as a heterodimeric partner, this receptor is known to be constitutively expressed in cells [53]; therefore, focusing on PPARs, we do not show the expression of RXR in this paper. Nevertheless, it has been reported that TR*β* and PPAR receptors can compete for binding to RXRs in the nucleus [54]. Since we have found PPRE within the sequence of TR*β*2-specific promoter, located in intron IV of* THRB* gene, the bidirectional regulation of TR*β*2 and PPARs is thought to be more complex. Results presented in the current manuscript indicated enhanced transactivation of TR*β*2 by all PPARs isoforms in response to the treatment with experimental FA (Table 2) that may be evidence of the functional activity of the TR*β*2-specific PPRE; however, this needs further studies. The most significant effect was measured for the synthetic CLA isomers, especially* trans10,cis12* (Table 2). Taken together, our findings showed that transcription levels of TR*β*2 are elevated by PPARs and their agonists. Simultaneously, TR*β*1 isoform has been shown to compete with PPAR for access to the RXR coreceptor or for PPRE binding sites in promoter regions of regulated genes [50] that could suggest TR*β*1-mediated inhibitory role in expression of TR*β*2 isoform and possibly other PPAR-responsive genes.

*WT1* gene, as a transcription factor, directly or indirectly interacts with a number of genes involved in cell cycle and neoplasia, including* HIF1A*,* AREG*,* SRY*,* NROB1*,* SOX9*,* IGF2*,* MDM4*,* BRCA1*,* TP53*, and* SP1* (NCBI Gene). Available literature suggests an oncogenic nature of* WT1* and has shown its overexpression in various tumors and tumor cell lines, especially in breast cancer cells and melanoma [55, 56]. In addition, decreased levels of* WT1* gene expression correlated with reduced cell proliferation in both melanoma and breast cancer cells [57, 58].* WT1* has also been linked with malignant transformation in breast cancer, and its overexpression associated with reduced susceptibility to drug treatment. Indeed, it has been shown for estrogen-dependent lines that* WT1* positively regulates the expression of* EGFR* and* HER2* [55], contributing to the resistance to hormone therapy [59, 60]. In melanoma, in vitro* WT1* silencing resulted in decreased cell proliferation, followed by apoptosis induction with caspase-3 activation [61], while in vivo it reduced the melanoma metastatic to lungs [56]. On the other hand, some studies indicate that pharmacologic activation of PPAR*δ* by its agonists (GW0742 and GW501516) inhibited proliferation of the murine melanoma cells, accompanied by downregulation of* WT1* [62]. It was suggested that PPAR*δ* can act via the PPRE in the* WT1* promoter and directly suppress its activity; however, our results do not support this hypothesis. Although the use of a known PPAR*δ* agonist, GW0742, resulted in PPAR*δ* activation (Figure 3(b)), no decrease in the expression of* WT1* was measured (Table 1). This contradiction may result from the use of different biological materials suggesting cell/tissue-specific regulation and/or association/dissociation of different corepressors or coactivators to transcription machinery. Interestingly, we showed that treatment with EFA-CLA and* cis9,trans11* reduced expression of* WT1* via the activation of PPAR*δ* (Table 2). A similar effect was observed for other experimental FA (Table 2) suggesting that various PPAR ligands may exert different effects in different cells; however, this hypothesis should be studied.

## 5. Conclusion

In conclusion, potential tumor suppressor properties of PPAR receptors make their ligands attractive candidates for the development of new chemopreventive, anticancer agents. Here, we show for the first time a functional food product, CLA-enriched egg (EFA-CLA), that is more effective in reducing of MCF-7 cancer cells proliferation than synthetic CLA isomers. This EFA-CLA effect could result from the high content of* cis9,trans11* isomer, altered SFA/MUFA ratio in enriched egg yolks, and/or supportive role of* trans10,cis11 *isomer in regulation of specific genes. Our results indicate that EFA-CLA can act as a ligand of PPARs, showing an agonist activity, specifically towards the PPAR*γ* isoform. Control, synthetic* cis9,trans11* isomer of CLA exerted an agonist effect on all PPAR receptors, while* trans10,cis12* showed no effects or even acted as an antagonist of PPAR*γ*. However, this isomer was able to regulate some specific genes containing PPREs such as* TCF20* involved in cell cycle arrest. Simultaneously,* cis9,trans11 *isomer upregulated* THRB* suppressor and downregulated* WT1* oncogene showing a small part of a PPAR action that in case of EFA-CLA leads to the observed reduction in proliferation of the breast cancer cells. It seems therefore that CLA-enriched eggs could be considered as food products with anticancer potential.

## Supplementary Material

Supplementary material for transfection control: Western blot and RT-PCR results, including nucleotide sequences of primers.
Supplementary material for quantitative verification of selected genes: Nucleotide sequences of primers.

## Figures and Tables

**Table 1:** Identification of in silico putative PPAR-responsive genes.

Gene symbol	Transcript	Position	NCBI reference sequence
*BCAR3*	AGGTCA**G**AGGTCA	93663502–93663514	NC_000001.11
*LZTS1*	AGGTCA**A**AGGTCA	20248971–20248983	NC_000008.11
*SLC5A1*	AGGTCA**C**AGGTCA	32033858–32033870	NC_000022.11
*TCF20*	AGGTCA**T**AGGTCA	42271609–42271621	NC_000022.11
*WT1*	AGGTCA**G**AGGTCA	32470961–32470973 32470822–32470834	NC_000011.10
*ZNF621*	AGGTCA**G**AGGTCA	41052623–41052635	NC_000003.12
*THRB* (TR*β*2)	AGGTCA**C**AGGTCA	24169753–24169765	NC_000003.12

*BCAR3*: breast cancer antiestrogen resistance 3; *LZTS1*: leucine zipper putative tumor suppressor 1;* SLC5A*: solute carrier family 5 member 1; *TCF20*: transcription factor 20; *WT1*: Wilms tumor 1; *ZNF621*: zinc finger protein 621; *THRB*: thyroid hormone receptor beta.

**Table 2:** mRNA expression of PPARs-responsive genes in PPAR-transfected MCF-7 cells (with overexpression of PPARs) after treatment with experimental FA or specific agonist/antagonist of PPAR for 48 h.

Gene symbol	FC values ± SD					
	EFA versus NC	EFA-CLA versus NC	*cis9,trans11CLA* versus NC	*trans10,cis12CLA *versus* NC*	Agonist versus NC	Antagonist versus NC
(A) mRNA expression of PPAR*α*-dependent genes						
*TCF-20*	−1.00^*∗*^ ± 0.02	1.02 ± 0.03	1.10 ± 0.24	1.70^*∗*^ ± 0.14	1.24 ± 0.14	−3.09^*∗*^ ± 0.23
*WT1*	−1.32 ± 0.11	−1.49^*∗*^ ± 0.11	−1,86^*∗*^ ± 0.07	−1.31 ± 0.23	1.34 ± 0.26	−2.11 ± 0.18
*ZNF621*	1.36 ± 0.16	1.80^*∗*^ ± 0.20	−2.51^*∗*^ ± 0.04	1.09 ± 0.19	−1.06 ± 0.18	−1.20 ± 0.24
*THRB* (TR*β*2)	2.49 ± 0.08	1.15 ± 0.12	1.74^*∗*^ ± 0.09	7.98^*∗*^ ± 0.34	2.54 ± 0.22	−1.77^*∗*^ ± 0.00

(B) mRNA expression of PPAR*δ*-dependent genes						
*TCF-20*	2.03^*∗*^ ± 0.04	3.08^*∗*^ ± 0.03	7.05^*∗*^ ± 0.11	13.02^*∗*^ ± 0.08	−1.43^*∗*^ ± 0.01	2.36 ± 0.08
*WT1*	1.18 ± 0.26	−1.38^*∗*^ ± 0.03	−1.52^*∗*^ ± 0.05	1.71 ± 0.29	1.90^*∗*^ ± 0.04	1.81^*∗*^ ± 0.01
*ZNF621*	1.37^*∗*^ ± 0.03	−1.29^*∗*^ ± 0.02	1.09 ± 0.16	−1.26 ± 0.19	−1.76 ± 0.11	−1.23^*∗*^ ± 0.03
*THRB* (TR*β*2)	1.61^*∗*^ ± 0.02	1.33^*∗*^ ± 0.01	6.67^*∗*^ ± 0.09	18.15^*∗*^ ± 0.11	1.90^*∗*^ ± 0.04	1.81^*∗*^ ± 0.01

(C) mRNA expression of PPAR*γ*-dependent genes						
*TCF-20*	2.09^*∗*^ ± 0.03	3.21^*∗*^ ± 0.04	6.66^*∗*^ ± 0.16	13.48^*∗*^ ± 0.09	1.92^*∗*^ ± 0.03	2.00^*∗*^ ± 0.01
*WT1*	−1.02 ± 0.04	−1.24 ± 0.06	−1.32^*∗*^ ± 0.03	−1.02 ± 0.08	1.48 ± 0.07	−1.47^*∗*^ ± 0.03
*ZNF621*	2.99^*∗*^ ± 0.01	3.12^*∗*^ ± 0.17	−1.13 ± 0.05	1.46 ± 0.07	5.97^*∗*^ ± 0.20	3.76^*∗*^ ± 0.10
*THRB* (TR*β*2)	1.09 ± 0.01	1.14 ± 0.01	9.96^*∗*^ ± 0.10	17.22^*∗*^ ± 0.13	−1.91^*∗*^ ± 0.01	−1.58^*∗*^ ± 0.00

FC: fold change; NC: negative control. Agonist/antagonist: for PPAR*α*, WY14643/GW-6471; for PPAR*δ*, GW-0742/GSK0660; for PPAR*γ*, troglitazone/T0070907. ^*∗*^*p* < 0.05.
