# Supplementary material for: Fatty Acids of CLA-Enriched Egg Yolks Can Induce Transcriptional Activation of Peroxisome Proliferator-Activated Receptors in MCF-7 Breast Cancer Cells
Source: PPAR Res. 2017 Mar 26;2017:2865283. doi: 10.1155/2017/2865283 (PMC5385215; doi:10.1155/2017/2865283)
Supplement: Supplementary file 1 — Supplementary material for transfection control: Western blot and RT-PCR results, including nucleotide sequences of primers. Supplementary material for quantitative verification of selected genes: Nucleotide sequences of primers. [file 2865283.f1.pdf]

S1 Figure. Control of transfection

A) PPARA

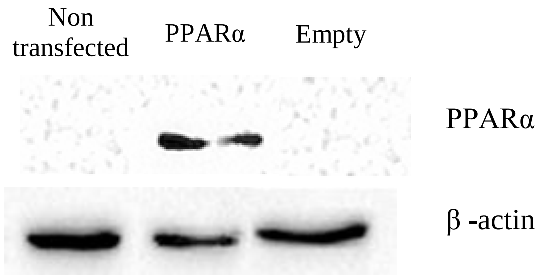

B) PPARD

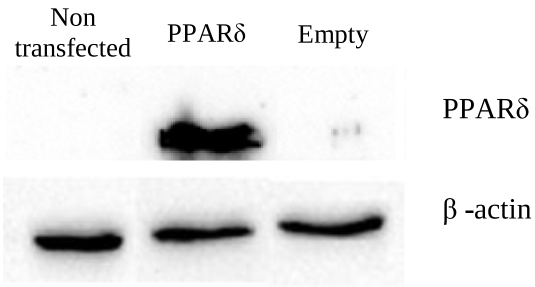

C) PPARG

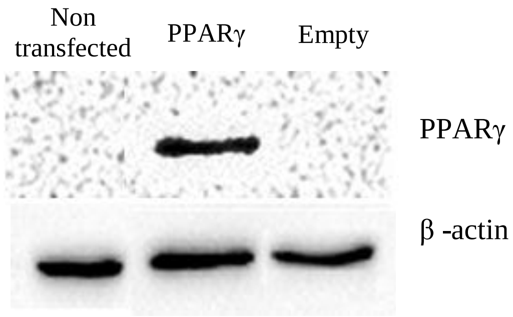

Non-transfected, wild MCF-7 cells; PPARα, MCF-7 cells transfected with pcDNA 6.2PPARα plasmid; PPARδ, MCF-7 cells transfected with pcDNA 6.2PPARδ plasmid; PPARγ, MCF-7 cells transfected with pcDNA 6.2PPARγ plasmid; Empty, MCF-7 cells transfected with pcDNA 6.2 plasmid; β –actin, actin beta.

S1 Table. Nucleotide sequences of primers

Gene	Primers forward 5’→3’	Primers reverse 5’→3’	NCBI Reference Sequence
PPARA	CGTTATCTGAAGAGTTCCTG	GTGAAAGCGTGTCCCGTGATG	NM_001001928.2
PPARD	CACATCTACAATGCCTACCTGA	CTGCCTGCCACAATGTCTCG	NM_001171820.1
PPARG	TGGAACTTGATGACAGTGACCT	TGTCAGAGCTCAGGCACAGT	NM_005037.5
TCF20	GAGCAGCCTTTGTTGTAGCAG	GGTTTGTGGCAGGCTCTATG	NM_005650
THRB (TRβ2)	TGCCCAGACCTTCCAAACG	AACCCTTGCAGCCTTCACAC	NM_001252634.1
WT1	CATGCTTGAATGAGTGGTTG	CGCAATCAGGGTTACAGCAC	NM_000378
ZNF621	GCATAATTCTCCAGCATCACC	ACCCAGAAGACAGTGCATGA	NM_001098414.2
ACTB	GCTGGGGTGTTGAAGGTCTC	CGGCATCGTCACCAACTG	NM_001101.3
GAPDH	ACCATCTTCCAGGAGCGAGA	GACTCCACGACGTACTCAGC	NM_001289746.1
HPRT1	CAGAGGGCTACAATGTGATG	TGGCGTCGTGATTAGTGATG	NM_000194.2
HSP90AB1	CCACTTGGCAGTCAAGCACT	CACACGGCGGACATAGAGTT	NM_001271972.1

ACTB, actin, beta; *GAPDH*, glyceraldehyde-3-phosphate dehydrogenase; *HPRT1*, hypoxanthine phosphoribosyltransferase 1; *HSP90AB1*, heat shock protein 90kDa alpha (cytosolic); *PPARA*, peroxisome proliferator activated receptor alpha; *PPARD*, peroxisome proliferator activated receptor delta; *PPARG*, peroxisome proliferator activated receptor gamma; *TCF20*, transcription factor 20; *THRB*, thyroid hormone receptor beta; *WT1*, wilms tumor 1; *ZNF621*, zinc finger protein 621;

S2 Table. **Control of transfections**

A)

		Plasmid control		Insert control	
**PPARA**	Cells	MCF-7	MCF-7 pcDNA6.2 PPARA	MCF-7	MCF-7 pcDNA6.2 PPARA
Primers	Forward	TTGGCACCAAAATCAACGG		ACAAGTGCCTGAGCGTGGG	
	Reverse	TCTCCAGGCGATCTGACGG		CTTCAGGTCGGCTGTCTCG	
	Cq value I	N/A	8,04	N/A	11,22
	Cq value II	N/A	10,31	N/A	7,04
	Cq (ACTB) I	20,83	21,81		
	Cq (ACTB) II	20,83	21,23		

B)

		Plasmid control		Insert control	
**PPARD**	Cells	MCF-7	MCF-7 pcDNA6.2 PPARD	MCF-7	MCF-7 pcDNA6.2 PPARD
Primers	Forward	TTGGCACCAAAATCAACGG		ACAAGTGCCTGAGCGTGGG	
	Reverse	TCTCCAGGCGATCTGACGG		CTTCAGGTCGGCTGTCTCG	
	Cq value I	N/A	8,67	N/A	5,68
	Cq value II	N/A	11,01	N/A	11,94
	Cq (ACTB) I	22,49	25,47		
	Cq (ACTB) II	22,19	25,01		

C)

		Plasmid control		Insert control	
**PPARG**	Cells	MCF-7	MCF-7 pcDNA6.2 PPARG	MCF-7	MCF-7 pcDNA6.2 PPARG
Primers	Forward	TTGGCACCAAAATCAACGG		CAGGGCTTCATGACCAGAGA	
	Reverse	TCTCCAGGCGATCTGACGG		GCTGTCGTCCAGTTCCAGG	
	Cq value I	N/A	9,14	N/A	5,65
	Cq value II	N/A	10,53	N/A	5,77
	Cq (ACTB) I	23,87	24,04		
	Cq (ACTB) II	23,9	24,1		

D)

Nucleotide sequences of primers

Target	Primers forward 5’→3’	Primers reverse 5’→3’
pPPARA-137-DNAiRNA	ACAAGTGCCTGAGCGTGGG	CTTCAGGTCGGCTGTCTCG
pPPARD-136-DNAiRNA	TGGAACTGGACGACAGCGA	TGCAGATGGAACTCGAGGG
pPPARG-114-DNAiRNA	CAGGGCTTCATGACCAGAGA	GCTGTCGTCCAGTTCCAGG
cDNA6-CMV-136-DNA	TTGGCACCAAAATCAACGG	TCTCCAGGCGATCTGACGG
